# Sexual relationship power, intimate partner violence and dyadic adjustment during pregnancy and affecting factors

**DOI:** 10.1590/1980-220X-REEUSP-2025-0019en

**Published:** 2025-05-09

**Authors:** Melek Balçık Çolak, Bihter Akın, Yasemin Erkal Aksoy

**Affiliations:** 1 Sakarya University, Health Sciences Faculty, Department of Midwifery, Sakarya, Turkey.; 2 Selcuk University, Health Sciences Faculty, Department of Midwifery, Konya, Turkey.

**Keywords:** Coitus, Intimate Partner Violence, Pregnancy, Coito, Violência por Parceiro Íntimo, Gravidez

## Abstract

**Objective:**

This study was conducted to investigate issues such as sexual relationship power, intimate partner violence and dyadic adjustment during pregnancy and affecting factors.

**Methods:**

The study data were collected by administering the Pregnant Women Information Form, Sexual Relationship Power Scale (SRPS), Intimate Partner Violence Attitude Scale (IPVAS)—Revised and Revised Dyadic Adjustment Scale (RDAS).

**Results:**

The mean scores the participating pregnant women obtained from the overall SRPS, IPVAS - Revised and RDAS were 3.06 ± 0.48, 49.03 ± 12.03 and 54.49 ± 8.17, respectively. While sexual relationship power was positively correlated with dyadic adjustment, intimate partner violence was negatively associated with dyadic adjustment. Factors influencing SRPS scores were education level, employment status, husband’s alcohol use, and exposure to physical violence. Factors affecting dyadic adjustment were husband’s education level, occupation, pregnancy trimester, number of pregnancies, sexual relationship power, and intimate partner violence attitudes.

**Conclusion:**

While the husbands’ characteristics such as education and profession affected the dyadic adjustment, the pregnant women’s attitudes towards dominance in sexual life and violence in the intimate relationships were the predictors of the dyadic adjustment. Based on these findings, interventions aimed at increasing women’s sexual relationship power and reducing intimate partner violence during pregnancy may support dyadic adjustment.

## RESUMO

**Objetivo:**

Este estudo foi realizado para investigar o poder no relacionamento sexual, a violência do parceiro íntimo e o ajuste diádico durante a gravidez, bem como os fatores que os influenciam.

**Métodos:**

Os dados do estudo foram coletados por meio da aplicação da Ficha de Informação da Grávida, da Escala de Poder nas Relações Sexuais (EPRS), da Escala Revisada de Atitudes em Relação à Violência do Parceiro Íntimo (ER-ARVPI) e da Escala Revisada de Ajustamento Diádico (ERAD).

**Resultados:**

As pontuações médias obtidas pelas grávidas no EPRS geral, no ER-ARVPI - ERAD foram, respetivamente, de 3,06 ± 0,48, 49,03 ± 12,03 e 54,49 ± 8,17. Verificou-se uma correlação positiva entre o poder no relacionamento sexual e o ajustamento diádico, ao passo que a violência do parceiro íntimo apresentou uma associação negativa com o ajustamento diádico. Os fatores que influenciaram as pontuações do incluíram o nível de escolaridade, a situação de emprego, o consumo de álcool pelo marido e a exposição à violência física. Os fatores que impactaram o ajuste diádico foram o nível de escolaridade do EPRS marido, a ocupação, o trimestre gestacional, o número de gestações, o poder no relacionamento sexual e as atitudes em relação à violência do parceiro íntimo.

**Conclusão:**

as características dos maridos, como a educação e a profissão, afetaram o ajuste diádico, ao passo que as atitudes das mulheres grávidas em relação à dominância na vida sexual e à violência nos relacionamentos íntimos foram os preditores do ajuste diádico. Com base nessas descobertas, é possível que intervenções que visem aumentar o poder das mulheres na vida sexual e reduzir a violência do parceiro íntimo durante a gravidez apoiem o ajuste diádico.

## INTRODUCTION

If both partners desire pregnancy and decide together, women’s satisfaction with their sexual life increases, leading to a healthier pregnancy^(1)^. In Turkish society, many women perceive sexuality as a duty for the man’s satisfaction or pregnancy, contributing to sexual dysfunctions^(2)^. Sexuality, being a multidimensional process influenced by psychosocial, cultural, behavioral, and clinical factors, may lead to sexual dysfunctions, adversely affecting women’s sexual lives and reproductive health^(3, 4)^.

A significant factor impacting women’s sexual life is dominance in sexual life, defined as the degree of control individuals have over sexual relationships and decision-making^(5)^. In a Canadian study conducted with female sex workers, it was demonstrated that decreased sexual desire increased the likelihood of intimate partner violence^(6)^. Violence against women remains one of the most widespread human rights violations, posing a major public health issue and a significant cause of death and disability among women aged 16–44 years^(7)^. The WHO reports that one in three women globally experience violence, and that 1–28% of them are subjected to physical violence during pregnancy^(8)^. Such violence can harm the woman and fetus, underscoring its importance in healthcare. Intimate partner violence includes physical, emotional, sexual, and economic abuse, as well as control over contraception or medical care^(11)^.

In Turkey, factors like place of residence, age, and spouses’ harmful habits influence domestic violence against women^(12)^. Pregnancy, a developmental crisis, significantly affects dyadic adjustment—encompassing happiness, satisfaction, and harmony in relationships^(13)^. Physical and psychological changes during pregnancy, particularly in the third trimester, can decrease sexual desire and cause postpartum sexual dysfunction^(14)^. These findings suggest links between dominance in sexual life, intimate partner violence, and dyadic adjustment. However, research in which these interrelated factors are addressed is limited. The present study was aimed at exploring factors influencing dominance in sexual life, intimate partner violence attitudes, dyadic adjustment in pregnant women, and relationships between relevant measures.

## METHODS

### Study Design and Sample

The present study was conducted online using Google Forms (https://forms.gle/DDde5HdN1oPeXLCi9) from August 2022 to March 2023. Surveys were shared via social media (Facebook, Instagram, WhatsApp), requiring email entry to prevent duplicate submissions. The participants provided informed consent by ticking a checkbox before completing the survey.

***Inclusion criteria***: having healthy pregnancy, being in the second or third trimester of pregnancy, being able to use social media tools and volunteering to participate in the study.

***Exclusion criteria***: having a high-risk pregnancy, having a chronic or psychiatric disease. Of the participants, those who did not fill out all of the forms during the data collection process were also excluded from the study. The population of the study consisted of pregnant women who volunteered to participate in the study across the country. The minimum sample size required to conduct the study was calculated as 384 using the sampling method for unknown population in the Epi Info 2022 software package at a prevalence rate of 50.0% and the confidence level of 95.0%. The study was completed with 389 pregnant women who were selected using the haphazard sampling method and who agreed to participate in the study and filled out the web-based data collection form.

### Data Collection Tools

The study data were collected using the Pregnant Women Information Form, Sexual Relationship Power Scale (SRPS), Intimate Partner Violence Attitude Scale (IPVAS) - Revised, and Revised Dyadic Adjustment Scale (RDAS). These tools were transferred to Google Forms, and the participants were invited via a shareable link using haphazard sampling. Informed consent was obtained on the first page of the form. It took each participant 15–20 minutes to fill in the forms.

Pregnant Women Information Form: This 14-item form, prepared based on the literature, is administered to collect information on the rspondent’s socio-demographic (e.g., age, education, profession, health insurance, income, and residence) and pregnancy-related characterisitics (e.g., the number of total pregnancies, miscarriages, and curettages)^(15)^.

Sexual Relationship Power Scale (SRPS): The SRPS developed by Pulerwitz et al.^(5)^ is used to measure the effectiveness of gender equality programs. The validity and reliability study of the Turkish version of the SRPS was conducted by Uçan and Baydur. The Cronbach’s alpha value is 0.85 for the relationship control subscale, and 0.76 for the decision-making dominance subscale^(16)^. Scores range from 23 to 92, with higher scores indicating greater power in relationships. The Revised Dyadic Adjustment Scale (RDAS) consists of 14 items and the following 3 dimensions: Satisfaction (4 items), Consensus (6 items), and Cohesion (4 items). The Cronbach’s alpha values for the present study were 0.89 for the overall scale, 0.88 for the Relationship Control subscale, and 0.79 for the Decision-Making Dominance subscale.

Intimate Partner Violence Attitude Scale (IPVAS)—Revised: The IPVAS developed by Fincham et al.^(17)^ and adapted into Turkish by Toplu-Demirtaş et al.^(18)^ is used to measure attitudes toward psychological and physical aggression. Scores range from 17 to 85, with higher scores reflecting more favorable attitudes toward aggression. The scale has 17 items and 3 dimensions. The minimum and maximum scores that can be obtained from the dimensions of violence, control, and abuse are 4 and 20, 6 and 30, and 7 and 35, respectively. the Cronbach’s alpha for the full IPVAS-R was 0.72. Internal consistency or Violence, Control and Abuse subscales were 0.72, 0.62, and 0.65 respectively^18^. The Cronbach’s alpha values were 0.82 for the overall scale, 0.86 for the Violence subscale, 0.60 for the Control subscale, and 0.86 for the Abuse subscale in the present study.

Revised Dyadic Adjustment Scale (RDAS): The RDAS adapted to Turkish by Bayraktaroğlu and Çakıcı, RDAS is administered to assess relationship quality. Scores range from 14 to 70, with higher scores indicating better quality. As a result of the scale’s validity analysis, it was determined that it comprises three dimensions—satisfaction, negotiation, and conflict—consisting of 14 items. The internal consistency coefficients for the total scale and its dimensions ranged between 0.74 and 0.87^(19, 20)^. The Cronbach’s alpha values were 0.82 for the overall scale, 0.87 for Satisfaction, 0.81 for Consensus, and 0.70 for Cohesion dimensions in the present study.

### Data Analysis

The study data were analyzed using the IBM SPSS (Statistical Package for the Social Sciences) Version 29.0. Descriptive data were presented as numbers, percentages, and means. P values less than 0.05 were considered significant. Normality was assessed with the Kolmogorov-Smirnov test. The independent samples t-test and ANOVA were used for group comparisons, with Post Hoc Tukey test for significant ANOVA results. Pearson correlation was used to assess relationships between the variables. Linear regression (Backward method) was used to analyze factors affecting SRPS, IPVAS, and RDAS scores through three models.

## RESULTS

The mean age of the pregnant women participating in the study was 28.49 ± 5.39 (min = 18, max = 48) years. The mean age of their husbands was 31.74 ± 5.38 (min = 21, max = 50) years. The mean scores the participating pregnant women obtained from the overall data collection tools were as follows: SRPS: 3.06 ± 0.48, IPVAS: 49.03 ± 12.03, and RDAS: 54.49 ± 8.17. Statistically significant differences were found in SRPS, IPVAS, and RDAS scores based on several variables. Mothers with higher education levels reported significantly higher SRPS and RDAS scores compared to those with lower education levels (p < 0.001). Employed mothers had significantly higher SRPS, IPVAS, and RDAS scores than unemployed mothers (p < 0.05). Husbands’ alcohol consumption was associated with significantly lower SRPS and RDAS scores (p < 0.01). Multiparous mothers had significantly lower SRPS and RDAS scores compared to primiparous mothers (p < 0.01). Additionally, experiencing physical violence from a spouse was significantly related to lower SRPS and RDAS scores (p < 0.001) (Table 1). According to the results of the correlation analysis, there was a positive significant relationship between the mean scores the participants obtained from the overall SRPS, and the overall RDAS and its subscales (r = 0.32 to 0.46).

**Table 1:** Comparison of the descriptive and obstetric characteristics of the participants and the mean scores they obtained from the overall SRPS, IPVAS and RDAS – Many provinces of Türkiye, 2023.

Variables	N(%)	SRPS Total Mean ± SD	IPVAS Total Mean ± SD	RDAS Total Mean ± SD
Participants’ Age Groups (Years)				
18–30	256(65.8)	3.09 ± 0.44	49.10 ± 11.22	55.07 ± 7.53
≥31	133(34.2)	3.02 ± 0.56	48.90 ± 13.50	53.39 ± 9.21
t		1.224	0.141	1.928
p		0.222	0.888	0.055
Husbands’ Age Groups (Years)				
18–30	184(47.3)	3.11 ± 0.43	48.66 ± 11.34	55.21 ± 7.55
≥31	205(52.7)	3.02 ± 0.52	49.36 ± 12.63	53.85 ± 8.66
t		1.758	–0.570	1.652
p		0.075	0.569	0.099
Participants’ Educational status				

Primary school graduates^a^	70(18.0)	2.76 ± 0.57	50.00 ± 12.04	51.44 ± 9.37
High school graduates^b^	143(36.8)	2.98 ± 0.51	48.06 ± 12.38	53.75 ± 7.61
Higher education graduates^c^	176(45.2)	3.25 ± 0.31	49.43 ± 11.74	56.31 ± 7.67
F		32.427	0.783	10.282
p		<0.001	0.458	<0.001
*Post Hoc Test*		c>b>a		c>a, b
Husbands’ educational status				
Primary school graduates^a^	64(16.5)	2.82 ± 0.63	48.59 ± 13.14	50.93 ± 9.34
High school graduates^b^	155(39.8)	3.02 ± 0.46	48.82 ± 11.39	54.40 ± 7.91
Higher education graduates^c^	170(43.7)	3.19 ± 0.39	49.39 ± 12.22	55.91 ± 7.54
F		15.551	0.142	8.998
p		<0.001	0.868	<0.001
*Post Hoc Test*		c>b>a		c, b>a
Participants’ Employment Status				
Not employed	221(56.8)	2.94 ± 0.52	47.89 ± 11.58	53.75 ± 8.43
Employed	168(43.2)	3.23 ± 0.38	50.53 ± 12.47	55.47 ± 7.73
t		–6.301	–2.153	–2.057
p		<0.001	0.032	0.040
Husband’s occupation				
Self-employed^a^	91(23.4)	2.96 ± 0.54	46.96 ± 12.49	53.09 ± 8.85
Government officer^b^	99(25.4)	3.14 ± 0.39	50.49 ± 13.22	54.09 ± 7.89
Worker^c^	169(43.4)	3.03 ± 0.51	49.73 ± 11.71	54.46 ± 7.90
Others^d^	30(7.7)	3.30 ± 0.27	46.53 ± 6.00	60.23 ± 6.11
F		5.066	2.023	6.126
p		0.002	0.110	<0.001
*Post Hoc Test*		d>c, a - b>a		d>a, b, c
Perceived monthly family income				
Income less than expenses (low)^a^	130(33,4)	2.92 ± 0.58	47.69 ± 12.47	52.93 ± 8.71
Income equal to expenses (moderate)^b^	193(49,6)	3.18 ± 0.36	48.74 ± 10.85	56.19 ± 6.73
Income more than expenses (high)^c^	66(17.0)	3.00 ± 0.51	52.53 ± 13.83	52.59 ± 9.84
F		12.151	3.700	8.657
p		<0.001	0.026	<0.001
*Post Hoc Test*		b>a	c>a	b>a, c
Place of residence				
City^a^	238(61.2)	3.13 ± 0.46	49.41 ± 13.11	54.32 ± 8.33
District^b^	119(30.6)	2.99 ± 0.50	48.79 ± 10.96	55.19 ± 7.99
Village / town^c^	32(8.2)	2.83 ± 0.51	47.09 ± 5.78	53.18 ± 7.63
F		7.089	0.557	0.895
p		<0.001	0.573	0.409
*Post Hoc Test*		a>b, c		
Husband’s smoking status				
Smoker	228(58.6)	3.04 ± 0.50	49.00 ± 11.77	54.20 ± 8.07
Nonsmoker	161(41.4)	3.09 ± 0.45	49.08 ± 12.43	54.91 ± 8.32
t		–0.892	–0.062	–0.845
p		0.373	0.951	0.399
Husband’s alcohol consumption				
Yes	56(14.4)	2.76 ± 0.60	48.00 ± 11.07	51.05 ± 9.52
No	333(85.6)	3.11 ± 0.44	49.21 ± 12.19	55.07 ± 7.79
t		–4.184	–0.696	–2.996
p		<0.001	0.487	0.004
Parity				
Primiparous	222(57.1)	3.13 ± 0.43	49.29 ± 11.89	55.69 ± 7.69
Multiparous	167(42.9)	2.97 ± 0.54	48.69 ± 12.24	52.89 ± 8.53
t		3.015	0.485	3.388
p		0.003	0.628	0.001
The number of pregnancies				
1^a^	193(49.6)	3.13 ± 0.43	49.15 ± 11.89	56.18 ± 7.70
2^b^	114(29.3)	3.09 ± 0.44	49.09 ± 11.69	53.45 ± 8.58
≥3^c^	82(21.1)	2.87 ± 0.59	48.67 ± 12.91	51.97 ± 7.85
F		8.313	0.048	9.301
p		<0.001	0.953	<0.001
*Post Hoc Test*		a, b>c		a>b, c
Gestational age				
Second trimester	73(18.8)	3.01 ± 0.60	48.64 ± 14.57	51.63 ± 9.51
Third trimester	316(81.2)	3.07 ± 0.45	49.12 ± 11.39	55.15 ± 7.69
t		–0.833	–0.265	–2.952
p		0.407	0.792	0.004
Is the pregnancy a planned one?				
Yes	276(71.0)	3.11 ± 0.46	48.94 ± 12.18	54.91 ± 8.26
No	113(29.0)	2.95 ± 0.52	49.24 ± 11.69	53.47 ± 7.89
t		2.850	–0.222	1.575
p		0.005	0.825	0.116
Being Subjected to Physical Violence Perpetrated by Her Spouse				
Yes	22(5.7)	2.46 ± 0.66	48.22 ± 14.69	43.95 ± 7.97
No	367(94.3)	3.10 ± 0.45	49.08 ± 11.87	55.12 ± 7.75
t		–4.412	–0.324	–6.555
p		<0.001	0.746	<0.001

As their SRPS scores increased so did their RDAS scores. There was a negative significant relationship between the mean scores the participants obtained from the overall IPVAS and the overall RDAS and its subscales (r = –0.10 to –0.20). Their IPVAS scores decreased as their RDAS scores increased (Table 2). In the model explaining 36% of the variance with linear regression analysis, the variables which affected the mean scores the participants obtained from the overall SRPS were as follows: education level (p < 0.001, CI = 0.076/0.200), working outside the home (p = 0.006, CI = 0.036/0.219), husband’s alcohol use (p < 0.001, CI = 0.137/0.363), being exposed to physical violence perpetrated by the husband (p = 0.001, CI = 0.112/0.470), and mean score for the overall RDAS (p < 0.001, CI = 0.014/0.025). In the model explaining 32% of the variance with linear regression analysis, the variables which affected the mean scores the participants obtained from the overall RDAS were as follows: husband’s education level (p = 0.030, CI = 0.106/2.075), husband’s occupation (p = 0.012, CI = 0.210/1.686), the number of pregnancies (p = 0.012, CI = –1.997/–0.245), pregnancy trimester (p < 0.001, CI = 1.526/5.038), and the mean scores for the overall SRPS (p < 0.001, CI = 5.635/8.594) and IPVAS (p < 0.001, CI = –0.210/–0.097) (Table 3).

**Table 2:** Comparison of the descriptive and obstetric characteristics of the participants and the mean scores they obtained from the overall SRPS, IPVAS and RDAS – Many provinces of Türkiye, 2023.

		RDAS Total	RDAS Sub-Dimensions			IPVAS Total	IPVAS Sub-Dimensions		
			Satisfaction	Consensus	Cohesion		Violence	Control	Abuse
SRPS Total	r	0.467*	0.406*	0.381*	0.327*	0.099	0.217*	0.162*	–0.078
SRPS Sub-Dimensions									
Relationship Control	r	0.457*	0.392*	0.376*	0.319*	0.079	0.218*	0.148*	–0.101**
Decision-Making Dominance	r	0.297*	0.274*	0.230*	0.207*	0.130**	0.116**	0.145*	0.047
IPVAS Total	r	–0.174*	–0.202*	–0.108**	–0.107**				
IPVAS Sub-Dimensions									
Violence	r	0.239*	0.220*	0.199*	0.136*				
Control	r	–0.083	–0.109**	–0.028	–0.083				
Abuse	r	–0.377*	–0.394*	–0.281*	–0.203*				

**Table 3:** Comparison of the descriptive and obstetric characteristics of the participants and the mean scores they obtained from the overall SRPS, IPVAS and RDAS – Many provinces of Türkiye, 2023.

	β	t	p	%95 CI	
Model 1: Factors Affecting the Mean Scores the Participants obtained from the overall SRPS					
Educational status	0.138	4.407	<0.001	0.076	0.200
Working Outside the Home	0.127	2.743	0.006	0.036	0.219
Husband’s Alcohol Consumption	0.250	4.356	<0.001	0.137	0.363
Whether the Pregnancy is the planned one	–0.073	–1.652	0.099	–0.160	0.014
Being Subjected to Physical Violence perpetrated by Her Husband	0.291	3.204	0.001	0.112	0.470
RDAS Total	0.019	7.356	<0.001	0.014	0.025
R: 0.607, R^2^: 0.368, Durbin-Watson: 2.007 (p < 0.001)					
Model 2: Factors Affecting the Mean Scores the Participants obtained from the overall IPVAS					
Working Outside the Home	2.495	2.009	0.045	0.053	4.936
Perceived Monthly Family Income	1.875	2.115	0.035	0.132	3.618
RDAS Total	–0.278	–3.793	<0.001	–0.422	–0.134
R: 0.240, R^2^: 0.058, Durbin-Watson: 1.709 (p < 0.001)					
Model 3: Factors Affecting the Mean Scores the Participants obtained from the overall RDAS					
Husband’s Educational Status	1.091	2.178	0.030	0.106	2.075
Husband’s Occupation	0.948	2.527	0.012	0.210	1.686
The number of pregnancies	–1.121	–2.517	0.012	–1.997	–0.245
Pregnancy trimester	3.282	3.675	<0.001	1.526	5.038
SRPS Total	7.115	9.457	<0.001	5.635	8.594
IPVAS Total	–0.154	–5.358	<0.001	–0.210	–0.097
R: 0.569, R^2^: 0.324, Durbin-Watson: 1.728 (p < 0.001)					

## DISCUSSION

In the present study, factors influencing the mean scores obtained from the SRPS, IPVAS, and RDAS, as well as the relationships between these scores were investigated. The participants’ mean scores were 3.06 ± 0.48 for SRPS, 49.03 ± 12.03 for IPVAS, and 54.49 ± 8.17 for RDAS. No significant relationship was determined between these scores and the participants’ or their husbands’ ages. However, of the participants, those who had higher education levels or those who perceived their income as moderate scored higher on SRPS and RDAS than did the others, while those with higher income levels scored higher on IPVAS. Women residing in city centers also had higher SRPS scores.

The findings align partially with literature suggesting that higher education promotes dyadic adjustment, though some differences may stem from cultural factors^(21)^. Women with lower education levels may exhibit greater marital harmony by adopting more accepting and submissive roles in marriage. Financial stress, a significant factor in intimate partner violence, disrupts dyadic harmony and communication^(22)^. Participants working outside the home scored higher on all scales, potentially reflecting improved financial empowerment. However, balancing work responsibilities and household duties, coupled with the added burden of pregnancy, may increase the risk of conflict and violence^(23)^.

Of the participants, those whose husbands consumed alcohol and those who had three or more pregnancies scored lower on SRPS and RDAS. Spousal alcohol use negatively affects family harmony and is associated with higher rates of domestic violence^(9, 24)^. Interestingly, women in their third trimester scored higher on RDAS, suggesting that improved dyadic adjustment due to increased spousal support during later stages of pregnancy^(25)^. Of the participants, 5.7% reported that they were exposed to physical violence perpetrated by their husbands, consistent with global studies showing significant violence prevalence during pregnancy^(1, 9, 10)^. Women exposed to physical violence scored lower on SRPS and RDAS, highlighting the detrimental effects of violence on relationship dynamics and sexual power. These findings emphasize the need for heightened sensitivity and support for pregnant women facing intimate partner violence, given the potential risks to maternal and fetal health^(9)^. Sexual relationship power positively correlated with dyadic adjustment indicators such as consensus, satisfaction, and cohesion. Regression analysis demonstrated that education level, employment status, exposure to physical violence, spousal alcohol use, and RDAS scores influenced SRPS levels. IPVAS scores were associated with employment, family income, and RDAS scores, while RDAS levels were affected by the variables such as spousal education, occupation, number of pregnancies, trimester, and SRPS and IPVAS scores. These results suggest that demographic, economic, and relational factors significantly shape intimate relationships during pregnancy^(22)^. Identifying at-risk women and implementing targeted interventions are essential for improving outcomes for both mothers and their unborn children. Future research should involve larger, multi-center samples to provide a comprehensive understanding of these dynamics.

### Strengths and Limitations

One of the strengths of the present study is its relatively large sample size, which enhances the generalizability of the findings. Additionally, the study addresses an important and sensitive topic by examining the relationships between sexual relationship power, intimate partner violence, and dyadic adjustment during pregnancy. The use of validated scales further strengthens the study’s reliability. However, several limitations should be acknowledged. First, the cross-sectional design does not allow for causal inferences. Second, the study was conducted using a web-based survey, which may introduce selection bias, as it excludes pregnant women with limited internet access or lower digital literacy. Lastly, self-reported data may be subject to social desirability bias, potentially affecting the accuracy of responses. Future studies should consider longitudinal designs and incorporate diverse data collection methods to improve the robustness of the findings.

## CONCLUSION

The present study revealed that factors such as education, income level, spousal alcohol use, and exposure to physical violence influenced dominance in sexual life, dyadic adjustment, and intimate partner violence during pregnancy. It emphasizes the importance of addressing dyadic adjustment, sexual health, and domestic violence during prenatal care to promote maternal well-being and healthy development of the baby. Future research should focus on larger, multi-center samples to provide a broader understanding of these issues.
