# Supplementary material for: Sexual relationship power, intimate partner violence and dyadic adjustment during pregnancy and affecting factors
Source: Rev Esc Enferm USP. 2025 May 9;59:e20250019. doi: 10.1590/1980-220X-REEUSP-2025-0019en (PMC12064085; doi:10.1590/1980-220X-REEUSP-2025-0019en)
Supplement: Translation

# Translation: Poder en la relación sexual, violencia de pareja y ajuste diádico durante el embarazo: factores influyentes

**DOI:** 10.1590/1980-220X-REEUSP-2025-0019es

**Authors:** Melek Balçık Çolak, Bihter Akın, Yasemin Erkal Aksoy

**Affiliations:** 1 Sakarya University, Health Sciences Faculty, Department of Midwifery, Sakarya, Turkey.; 2 Selcuk University, Health Sciences Faculty, Department of Midwifery, Konya, Turkey.

**Keywords:** Coito, Violencia de Pareja, Embarazo

## Abstract

**Objetivo:**

Este estudio se llevó a cabo para investigar el poder en las relaciones sexuales, la violencia de pareja y el ajuste diádico durante el embarazo, así como los factores que los influyen.

**Métodos:**

Los datos del estudio se recopilaron mediante la aplicación del Formulario de Información para Mujeres Embarazadas, Escala de Poder en las Relaciones Sexuales (EPRS), La Escala Revisada de Actitudes ante la Violencia de Pareja (ERAVP), La Escala Revisada de Ajuste Diádico (ERAD).

**Resultados:**

Las puntuaciones medias obtenidas por las mujeres embarazadas en la EPRS general, la ERAVP, ERAD fueron de 3,06 ± 0,48, 49,03 ± 12,03 y 54,49 ± 8,17, respectivamente. Se observó una correlación positiva entre el poder en las relaciones sexuales y el ajuste diádico, mientras que la violencia de pareja mostró una asociación negativa con el ajuste diádico. Los factores que influyeron en las puntuaciones de la EPRS incluyeron el nivel educativo, la situación laboral, el consumo de alcohol del marido y la exposición a la violencia física. Los factores que impactaron el ajuste diádico fueron el nivel educativo del marido, la ocupación, el trimestre gestacional, el número de embarazos, el poder en las relaciones sexuales y las actitudes hacia la violencia de pareja.

**Conclusión:**

Las características de los maridos, como el nivel educativo y la profesión, afectaron el ajuste diádico, mientras que las actitudes de las mujeres embarazadas hacia la dominancia en la vida sexual y la violencia en las relaciones íntimas fueron predictores del ajuste diádico. Con base en estos hallazgos, es posible que las intervenciones dirigidas a aumentar el poder sexual de las mujeres y reducir la violencia de pareja durante el embarazo puedan favorecer el ajuste diádico.

## INTRODUCCIÓN

Si ambos miembros de la pareja desean un embarazo y lo deciden juntos, la satisfacción de la mujer con su vida sexual aumenta, lo que conduce a un embarazo más saludable^(1)^. En la sociedad turca, muchas mujeres perciben la sexualidad como un deber para la satisfacción del hombre o el embarazo, lo que contribuye a las disfunciones sexuales^(2)^. La sexualidad, al ser un proceso multidimensional influenciado por factores psicosociales, culturales, conductuales y clínicos, puede conducir a disfunciones sexuales, afectando negativamente la vida sexual y la salud reproductiva de la mujer^(3, 4)^. Un factor significativo que afecta a la vida sexual de las mujeres es el dominio en la vida sexual, definido como el grado de control que tienen las personas sobre las relaciones sexuales y la toma de decisiones^(5)^. En un estudio canadiense realizado con trabajadoras sexuales, se demostró que la disminución del deseo sexual aumentaba la probabilidad de violencia de pareja^(6)^. La violencia contra las mujeres sigue siendo una de las violaciones de los derechos humanos más extendidas, lo que supone un importante problema de salud pública y una causa significativa de muerte y discapacidad entre las mujeres de 16 a 44 años^(7)^. La OMS informa de que una de cada tres mujeres en todo el mundo sufre violencia, y que entre el 1 y el 28 % de ellas son objeto de violencia física durante el embarazo^(8)^. Esta violencia puede perjudicar a la mujer y al feto, lo que subraya su importancia en la atención sanitaria. La violencia de pareja incluye el abuso físico, emocional, sexual y económico, así como el control sobre la anticoncepción o la atención médica^(11)^. En Turquía, factores como el lugar de residencia, la edad y los hábitos nocivos de los cónyuges influyen en la violencia doméstica contra las mujeres^(12)^. El embarazo, una crisis de desarrollo, afecta significativamente al ajuste diádico, que abarca la felicidad, la satisfacción y la armonía en las relaciones^(13)^. Los cambios físicos y psicológicos durante el embarazo, especialmente en el tercer trimestre, pueden disminuir el deseo sexual y causar disfunción sexual posparto^(14)^. Estos hallazgos sugieren vínculos entre el dominio en la vida sexual, la violencia de pareja y el ajuste diádico. Sin embargo, la investigación en la que se abordan estos factores interrelacionados es limitada. El presente estudio tuvo como objetivo explorar los factores que influyen en el dominio en la vida sexual, las actitudes de violencia de pareja, el ajuste diádico en mujeres embarazadas y las relaciones entre las medidas relevantes.

## MÉTODOS

### Diseño del Estudio y Muestra

El presente estudio se llevó a cabo en línea utilizando Google Forms (https://forms.gle/DDde5HdN1oPeXLCi9) desde agosto de 2022 hasta marzo de 2023. Las encuestas se compartieron a través de las redes sociales (Facebook, Instagram, WhatsApp), requiriendo la entrada de correo electrónico para evitar envíos duplicados. Los participantes dieron su consentimiento informado marcando una casilla de verificación antes de completar la encuesta.Criterios de inclusión: tener un embarazo saludable, estar en el segundo o tercer trimestre de embarazo, poder utilizar herramientas de redes sociales y ofrecerse como voluntaria para participar en el estudio.Criterios de exclusión: tener un embarazo de alto riesgo, tener una enfermedad crónica o psiquiátrica. De las participantes, también se excluyó del estudio a aquellas que no completaron todos los formularios durante el proceso de recolección de datos. La población del estudio consistió en mujeres embarazadas que se ofrecieron como voluntarias para participar en el estudio en todo el país. El tamaño mínimo de la muestra requerido para llevar a cabo el estudio se calculó en 384 utilizando el método de muestreo para población desconocida en el paquete de software Epi Info 2022, con una tasa de prevalencia del 50,0 % y un nivel de confianza del 95,0 %. El estudio se completó con 389 mujeres embarazadas que fueron seleccionadas utilizando el método de muestreo aleatorio y que aceptaron participar en el estudio y rellenaron el formulario de recopilación de datos en línea.

### Herramientas de Recolección de Datos

Los daos del estudio se recolectaron utilizando el Formulario de Información para Mujeres Embarazadas, la Escala de Poder en las Relaciones Sexuales (Sexual Relationship Power Scale - SRPS en la sigla en inglés), la Escala de Actitud ante la Violencia de la Pareja Íntima (Intimate Partner Violence Attitude Scale - IPVAS en la sigla en inglés) - Revisada, y la Escala Revisada de Ajuste Diádico (Revised Dyadic Adjustment Scale - RDAS en la sigla en inglés). Estas herramientas se transfirieron a Google Forms, y se invitó a las participantes a través de un enlace compartible utilizando un muestreo aleatorio.

El consentimiento informado se obtuvo en la primera página del formulario. Cada participante tardó entre 15 y 20 minutos en rellenar los formularios.Formulario de información para mujeres embarazadas: Este formulario de 14 ítems, preparado en base a la literatura, se administra para recolectar información sobre las características sociodemográficas (por ejemplo, edad, educación, profesión, seguro médico, ingresos y residencia) y relacionadas con el embarazo (por ejemplo, el número total de embarazos, abortos espontáneos y legrados) de la encuestada^(15)^. Escala de Poder en las Relaciones Sexuales (SRPS): La SRPS desarrollada por Pulerwitz et al.^(5)^ se utiliza para medir la eficacia de los programas de igualdad de género. El estudio de validez y fiabilidad de la versión turca de la SRPS fue realizado por Uçan y Baydur. El valor alfa de Cronbach es de 0,85 para la subescala de control de la relación y de 0,76 para la subescala de dominio en la toma de decisiones^(16)^. Las puntuaciones oscilan entre 23 y 92, y las puntuaciones más altas indican un mayor poder en las relaciones. La Escala Revisada de Ajuste Diádico (RDAS) consta de 14 ítems y las siguientes 3 dimensiones: Satisfacción (4 ítems), Consenso (6 ítems) y Cohesión (4 ítems). Los valores alfa de Cronbach para el presente estudio fueron de 0,89 para la escala general, 0,88 para la subescala de Control de Relaciones y 0,79 para la subescala de Dominio en la Toma de Decisiones.Escala de Actitud ante la Violencia de la Pareja Íntima (IPVAS): revisada: La IPVAS desarrollada por Fincham et al.^(17)^ y adaptada al turco por Toplu-Demirtaş et al.^(18)^ se utiliza para medir las actitudes hacia la agresión psicológica y física. Las puntuaciones van de 17 a 85, y las puntuaciones más altas reflejan actitudes más favorables hacia la agresión. La escala tiene 17 ítems y 3 dimensiones. Las puntuaciones mínima y máxima que se pueden obtener de las dimensiones de violencia, control y abuso son 4 y 20, 6 y 30, y 7 y 35, respectivamente. El alfa de Cronbach para el IPVAS-R completo fue de 0,72. La consistencia interna o las subescalas de violencia, control y abuso fueron de 0,72, 0,62 y 0,65, respectivamente, 18. Los valores alfa de Cronbach fueron de 0,82 para la escala general, 0,86 para la subescala de violencia, 0,60 para la subescala de control y 0,86 para la subescala de abuso en el presente estudio.Escala de ajuste diádico revisada (RDAS): La RDAS adaptada al turco por Bayraktaroğlu y Çakıcı, se administra para evaluar la calidad de la relación. Las puntuaciones van de 14 a 70, y las puntuaciones más altas indican una mejor calidad. Como resultado del análisis de validez de la escala, se determinó que comprende tres dimensiones (satisfacción, negociación y conflicto) que constan de 14 ítems. Los coeficientes de consistencia interna para la escala total y sus dimensiones oscilaron entre 0,74 y 0,87^(19, 20)^. Los valores alfa de Cronbach fueron de 0,82 para la escala general, 0,87 para Satisfacción, 0,81 para Consenso y 0,70 para Cohesión en el presente estudio.

### Análisis de Datos

Los datos del estudio se analizaron utilizando el IBM SPSS (Statistical Package for the Social Sciences) versión 29.0. Los datos descriptivos se presentaron como números, porcentajes y medias. Los valores p inferiores a 0,05 se consideraron significativos. La normalidad se evaluó con la prueba de Kolmogorov-Smirnov. Se utilizaron la prueba t de muestras independientes y el ANOVA para las comparaciones de grupos, con la prueba post hoc de Tukey para los resultados significativos del ANOVA. Se utilizó la correlación de Pearson para evaluar las relaciones entre las variables. Se utilizó la regresión lineal (Backward method) para analizar los factores que afectan a las puntuaciones de SRPS, IPVAS y RDAS a través de tres modelos.

## RESULTADOS

La edad media de las mujeres embarazadas que participaron en el estudio fue de 28,49 ± 5,39 (mín. = 18, máx. = 48) años. La edad media de sus maridos fue de 31,74 ± 5,38 (mín. = 21, máx. = 50) años. Las puntuaciones medias que obtuvieron las mujeres embarazadas participantes en las herramientas generales de recopilación de datos fueron las siguientes: SRPS: 3,06 ± 0,48, IPVAS: 49,03 ± 12,03 y RDAS: 54,49 ± 8,17 (Tabla 1). Según los resultados del análisis de correlación, existía una relación positiva significativa entre las puntuaciones medias que las participantes obtuvieron en el SRPS general y el RDAS general y sus subescalas (r = 0,32 a 0,46).

**Tabla 1:** Comparación de las características descriptivas y obstétricas de las participantes y las puntuaciones medias que obtuvieron de la SRPS general, IPVAS y RDAS – Muchas provincias de Turquia, 2023.

Variables	N (%)	SRPS Total Media ± DE	IPVAS Total Media ± DE	RDAS Total Media ± DE
Grupos de Edad de los Participantes (Años)				
18–30	256(65,8)	3,09 ± 0,44	49,10 ± 11,22	55,07 ± 7,53
≥31	133(34,2)	3,02 ± 0,56	48,90 ± 13,50	53,39 ± 9,21
t		1,224	0,141	1,928
p		0,222	0,888	0,055
Grupos de Edad de los Esposos (Años)				
18–30	184(47,3)	3,11 ± 0,43	48,66 ± 11,34	55,21 ± 7,55
≥31	205(52,7)	3,02 ± 0,52	49,36 ± 12,63	53,85 ± 8,66
t		1,758	–0,570	1,652
p		0,075	0,569	0,099
Nivel Educativo de los Participantes				
Primaria completa^a^	70(18,0)	2,76 ± 0,57	50,00 ± 12,04	51,44 ± 9,37
Secundaria completa^b^	143(36,8)	2,98 ± 0,51	48,06 ± 12,38	53,75 ± 7,61
Educación superior completa^c^	176(45,2)	3,25 ± 0,31	49,43 ± 11,74	56,31 ± 7,67
F		32,427	0,783	10,282
p		<0,001	0,458	<0,001
*Post Hoc Test*		c>b>a		c>a, b
Nivel Educativo de los Esposos				
Primaria completa^a^	64(16,5)	2,82 ± 0,63	48,59 ± 13,14	50,93 ± 9,34
Secundaria completa^b^	155(39,8)	3,02 ± 0,46	48,82 ± 11,39	54,40 ± 7,91
Educación superior completa^c^	170(43,7)	3,19 ± 0,39	49,39 ± 12,22	55,91 ± 7,54
F		15,551	0,142	8,998
p		<0,001	0,868	<0,001
*Post Hoc Test*		c>b>a		c, b>a
Situación Laboral de los Participantes				
Desempleado	221(56,8)	2,94 ± 0,52	47,89 ± 11,58	53,75 ± 8,43
Empleado	168(43,2)	3,23 ± 0,38	50,53 ± 12,47	55,47 ± 7,73
t		–6,301	–2,153	–2,057
p		<0,001	0,032	0,040
Ocupación del Esposo				
Autónomo^a^	91(23,4)	2,96 ± 0,54	46,96 ± 12,49	53,09 ± 8,85
Empleado del gobierno^b^	99(25,4)	3,14 ± 0,39	50,49 ± 13,22	54,09 ± 7,89
Trabajador ^c^	169(43,4)	3,03 ± 0,51	49,73 ± 11,71	54,46 ± 7,90
Otros^d^	30(7,7)	3,30 ± 0,27	46,53 ± 6,00	60,23 ± 6,11
F		5,066	2,023	6,126
p		0,002	0,110	<0,001
*Post Hoc Test*		d>c, a - b>a		d>a, b, c
Ingreso Familiar Mensual Percibido				
Ingresos menores que gastos (bajos)^a^	130(33,4)	2,92 ± 0,58	47,69 ± 12,47	52,93 ± 8,71
Ingresos iguales a gastos (moderados)^b^	193(49,6)	3,18 ± 0,36	48,74 ± 10,85	56,19 ± 6,73
Ingresos mayores que gastos (h)^c^	66(17,0)	3,00 ± 0,51	52,53 ± 13,83	52,59 ± 9,84
F		12,151	3,700	8,657
p		<0,001	0,026	<0,001
*Post Hoc Test*		b>a	c>a	b>a, c
Lugar de Residencia				
Ciudad^a^	238(61,2)	3,13 ± 0,46	49,41 ± 13,11	54,32 ± 8,33
Distrito^b^	119(30,6)	2,99 ± 0,50	48,79 ± 10,96	55,19 ± 7,99
Villa / pueblo^c^	32(8,2)	2,83 ± 0,51	47,09 ± 5,78	53,18 ± 7,63
F		7,089	0,557	0,895
p		<0,001	0,573	0,409
*Post Hoc Test*		a>b, c		
Estado de Tabaquismo del Esposo				
Fumador	228(58,6)	3,04 ± 0,50	49,00 ± 11,77	54,20 ± 8,07
No Fumador	161(41,4)	3,09 ± 0,45	49,08 ± 12,43	54,91 ± 8,32
t		–0,892	–0,062	–0,845
p		0,373	0,951	0,399
Consumo de Alcohol del Esposo				
Si	56(14,4)	2,76 ± 0,60	48,00 ± 11,07	51,05 ± 9,52
No	333(85,6)	3,11 ± 0,44	49,21 ± 12,19	55,07 ± 7,79
t		–4,184	–0,696	–2,996
p		<0,001	0,487	0,004
Paridad				
Primípara	222(57,1)	3,13 ± 0,43	49,29 ± 11,89	55,69 ± 7,69
Multípara	167(42,9)	2,97 ± 0,54	48,69 ± 12,24	52,89 ± 8,53
t		3,015	0,485	3,388
p		0,003	0,628	0,001
Número de embarazos				
1^a^	193(49,6)	3,13 ± 0,43	49,15 ± 11,89	56,18 ± 7,70
2^b^	114(29,3)	3,09 ± 0,44	49,09 ± 11,69	53,45 ± 8,58
≥3^c^	82(21,1)	2,87 ± 0,59	48,67 ± 12,91	51,97 ± 7,85
F		8,313	0,048	9,301
p		<0,001	0,953	<0,001
*Post Hoc Test*		a, b>c		a>b, c
Edad Gestacional				
Segundo trimestre	73(18,8)	3,01 ± 0,60	48,64 ± 14,57	51,63 ± 9,51
Tercer trimestre	316(81,2)	3,07 ± 0,45	49,12 ± 11,39	55,15 ± 7,69
t		–0,833	–0,265	–2,952
p		0,407	0,792	0,004
¿El embarazo fue planificado?
Si	276(71,0)	3,11 ± 0,46	48,94 ± 12,18	54,91 ± 8,26
No	113(29,0)	2,95 ± 0,52	49,24 ± 11,69	53,47 ± 7,89
t		2,850	–0,222	1,575
p		0,005	0,825	0,116
Sufrió Violencia Física Perpetrada por su Esposo				
Si	22(5,7)	2,46 ± 0,66	48,22 ± 14,69	43,95 ± 7,97
No	367(94,3)	3,10 ± 0,45	49,08 ± 11,87	55,12 ± 7,75
t		–4,412	–0,324	–6,555
p		<0,001	0,746	<0,001

A medida que aumentaban sus puntuaciones en el SRPS, también lo hacían sus puntuaciones en el RDAS. Existía una relación negativa significativa entre las puntuaciones medias que los participantes obtuvieron en el IPVAS general y en el RDAS general y sus subescalas (r = –0,10 a –0,20). Sus puntuaciones en el IPVAS disminuyeron a medida que aumentaban sus puntuaciones en el RDAS (Tabla 2). En el modelo que explica el 36 % de la varianza con el análisis de regresión lineal, las variables que afectaron a las puntuaciones medias que los participantes obtuvieron en el SRPS general fueron las siguientes: nivel de educación (p < 0,001, IC = 0,076/0,200), trabajar fuera de casa (p = 0,006, IC = 0,036/0,219), consumo de alcohol del marido (p < 0,001, IC = 0,137/0,363), estar expuesta a violencia física perpetrada por el marido (p = 0,001, IC = 0,112/0,470) y puntuación media en el RDAS general (p < 0,001, IC = 0,014/0,025). En el modelo que explica el 32 % de la varianza con el análisis de regresión lineal, las variables que afectaron a las puntuaciones medias que los participantes obtuvieron en el RDAS general fueron las siguientes: nivel de educación del marido (p = 0,030, IC = 0,106/2,075), ocupación del marido (p = 0,012, IC = 0,210/1,686), número de embarazos (p = 0,012, IC = –1,997/–0,245), trimestre del embarazo (p < 0,001, IC = 1,526/5,038), y las puntuaciones medias para el SRPS general (p < 0,001, IC = 5,635/8,594) y el IPVAS (p < 0,001, IC = –0,210/–0,097) (Tabla 3).

**Tabla 2:** Comparación de las características descriptivas y obstétricas de las participantes y las puntuaciones medias que obtuvieron de la SRPS general, IPVAS y RDAS – Muchas provincias de Turquia, 2023.

		RDAS Total	Subdimensiones RDAS			IPVAS Total	Subdimensiones IPVAS		
			Satisfacción	Consensos	Cohesión		Violencia	Control	Abuso
SRPS Total	r	0,467*	0,406*	0,381*	0,327*	0,099	0,217*	0,162*	–0,078
Subdimensiones SRPS									
Control de la relación	r	0,457*	0,392*	0,376*	0,319*	0,079	0,218*	0,148*	–0,101**
Dominio de las decisiones	r	0,297*	0,274*	0,230*	0,207*	0,130**	0,116**	0,145*	0,047
IPVAS Total	r	–0,174*	–0,202*	–0,108**	–0,107**				
Subdimensiones IPVAS									
Violencia	r	0,239*	0,220*	0,199*	0,136*				
Control	r	–0,083	–0,109**	–0,028	–0,083				
Abuso	r	–0,377*	–0,394*	–0,281*	–0,203*				

**Tabla 3:** Comparación de las características descriptivas y obstétricas de las participantes y las puntuaciones medias que obtuvieron de la SRPS general, IPVAS y RDAS – Muchas provincias de Turquia, 2023.

	β	t	p	%95 IC	
Modelo 1: Factores que afectan las puntuaciones medias que obtuvieron los participantes del SRPS general					
Nivel Educativo	0,138	4,407	<0,001	0,076	0,200
Trabajo fuera de casa	0,127	2,743	0,006	0,036	0,219
Consumo de Alcohol del Esposo	0,250	4,356	<0,001	0,137	0,363
Si el embarazo fue planificado	–0,073	–1,652	0,099	–0,160	0,014
Ser sometida a violencia física perpetrada por su marido	0,291	3,204	0,001	0,112	0,470
RDAS Total	0,019	7,356	<0,001	0,014	0,025
R: 0,607, R2: 0,368, Durbin-Watson: 2,007 (p<0,001)					
Modelo 2: Factores que afectan las puntuaciones medias que obtuvieron los participantes del IPVAS general					
Trabajo fuera de casa	2,495	2,009	0,045	0,053	4,936
Ingreso Familiar Mensual Percibido	1,875	2,115	0,035	0,132	3,618
RDAS Total	–0,278	–3,793	<0,001	–0,422	–0,134
R: 0,240, R^2^: 0,058, Durbin-Watson: 1,709 (p<0,001)					
Modelo 3: Factores que afectan las puntuaciones medias que obtuvieron los participantes del RDAS general					
Nivel Educativo de los Esposos	1,091	2,178	0,030	0,106	2,075
Ocupación del Esposo	0,948	2,527	0,012	0,210	1,686
Número de embarazos	–1,121	–2,517	0,012	–1,997	–0,245
Trimestre de la gestación	3,282	3,675	<0,001	1,526	5,038
SRPS Total	7,115	9,457	<0,001	5,635	8,594
IPVAS Total	–0,154	–5,358	<0,001	–0,210	–0,097
R: 0,569, R^2^: 0,324, Durbin-Watson: 1,728 (p < 0,001)					

## DISCUSIÓN

En el presente estudio, se investigaron los factores que influyen en las puntuaciones medias obtenidas en el SRPS, el IPVAS y el RDAS, así como las relaciones entre estas puntuaciones. Las puntuaciones medias de los participantes fueron de 3,06 ± 0,48 para el SRPS, 49,03 ± 12,03 para el IPVAS y 54,49 ± 8,17 para el RDAS. No se determinó ninguna relación significativa entre estas puntuaciones y las edades de las participantes o de sus maridos. Sin embargo, de las participantes, aquellas que tenían niveles de educación más altos o aquellas que percibían sus ingresos como moderados obtuvieron puntuaciones más altas en SRPS y RDAS que las demás, mientras que aquellas con niveles de ingresos más altos obtuvieron puntuaciones más altas en IPVAS. Las mujeres que residían en centros urbanos también obtuvieron puntuaciones más altas en SRPS.

Los resultados coinciden en parte con la literatura que sugiere que la educación superior promueve el ajuste diádico, aunque algunas diferencias pueden deberse a factores culturales ^(21)^. Las mujeres con niveles de educación más bajos pueden mostrar una mayor armonía conyugal al adoptar roles más tolerantes y sumisos en el matrimonio. El estrés financiero, un factor significativo en la violencia de pareja, altera la armonía y la comunicación diádicas^(22)^. Las participantes que trabajaban fuera de casa obtuvieron puntuaciones más altas en todas las escalas, lo que podría reflejar una mayor autonomía económica. Sin embargo, el equilibrio entre las responsabilidades laborales y las tareas domésticas, junto con la carga añadida del embarazo, puede aumentar el riesgo de conflicto y violencia^(23)^.

De las participantes, aquellas cuyos maridos consumían alcohol y las que tuvieron tres o más embarazos obtuvieron puntuaciones más bajas en SRPS y RDAS. El consumo de alcohol por parte del cónyuge afecta negativamente a la armonía familiar y se asocia con mayores tasas de violencia doméstica^(9, 24)^. Curiosamente, las mujeres en su tercer trimestre obtuvieron puntuaciones más altas en RDAS, lo que sugiere una mejor adaptación diádica debido al aumento del apoyo conyugal durante las últimas etapas del embarazo^(25)^. De las participantes, el 5,7 % informó de que habían sido expuestas a violencia física por parte de sus maridos, lo que concuerda con estudios globales que muestran una prevalencia significativa de la violencia durante el embarazo^(1, 9, 10)^. Las mujeres expuestas a violencia física obtuvieron puntuaciones más bajas en SRPS y RDAS, lo que pone de relieve los efectos perjudiciales de la violencia en la dinámica de la relación y el poder sexual. Estos resultados subrayan la necesidad de aumentar la sensibilidad y el apoyo a las mujeres embarazadas que se enfrentan a la violencia de pareja, dados los riesgos potenciales para la salud materna y fetal^(9)^. El poder en las relaciones sexuales se correlacionó positivamente con indicadores de ajuste diádico como el consenso, la satisfacción y la cohesión. El análisis de regresión demostró que el nivel de educación, la situación laboral, la exposición a la violencia física, el consumo de alcohol por parte del cónyuge y las puntuaciones del RDAS influyeron en los niveles de SRPS. Las puntuaciones del IPVAS se asociaron con el empleo, los ingresos familiares y las puntuaciones del RDAS, mientras que los niveles del RDAS se vieron afectados por variables como la educación del cónyuge, la ocupación, el número de embarazos, el trimestre y las puntuaciones del SRPS y el IPVAS. Estos resultados sugieren que los factores demográficos, económicos y relacionales configuran significativamente las relaciones íntimas durante el embarazo^(22)^. Identificar a las mujeres en situación de riesgo y poner en marcha intervenciones específicas es esencial para mejorar los resultados tanto para las madres como para sus hijos por nacer. Las futuras investigaciones deberían incluir muestras multicéntricas más amplias para comprender en profundidad estas dinámicas.

### Fortalezas y Limitaciones

Uno de los puntos fuertes del presente estudio es su tamaño de muestra relativamente grande, lo que mejora la generalización de los resultados. Además, el estudio aborda un tema importante y delicado al examinar las relaciones entre el poder en las relaciones sexuales, la violencia de pareja y el ajuste diádico durante el embarazo. El uso de escalas validadas refuerza aún más la fiabilidad del estudio. Sin embargo, hay que reconocer varias limitaciones. En primer lugar, el diseño transversal no permite inferencias causales. En segundo lugar, el estudio se llevó a cabo mediante una encuesta en línea, lo que puede introducir un sesgo de selección, ya que excluye a las mujeres embarazadas con acceso limitado a Internet o con menor alfabetización digital. Por último, los datos autoinformados pueden estar sujetos a un sesgo de deseabilidad social, lo que puede afectar a la precisión de las respuestas. Los estudios futuros deberían considerar diseños longitudinales e incorporar diversos métodos de recopilación de datos para mejorar la solidez de los resultados.

## CONCLUSIONES

El presente estudio reveló que factores como la educación, el nivel de ingresos, el consumo de alcohol por parte del cónyuge y la exposición a la violencia física influyen en el dominio de la vida sexual, el ajuste diádico y la violencia de pareja durante el embarazo. Destaca la importancia de abordar el ajuste diádico, la salud sexual y la violencia doméstica durante la atención prenatal para promover el bienestar materno y el desarrollo saludable del bebé. Las investigaciones futuras deberían centrarse en muestras multicéntricas más amplias para proporcionar una comprensión más amplia de estas cuestiones.
